# Supplementary material for: Translation in Bacillus subtilis is spatially and temporally coordinated during sporulation
Source: Nat Commun. 2024 Aug 21;15:7188. doi: 10.1038/s41467-024-51654-6 (PMC11339384; doi:10.1038/s41467-024-51654-6)
Supplement: Supplementary file 1 — Supplementary Information [file 41467_2024_51654_MOESM1_ESM.pdf]

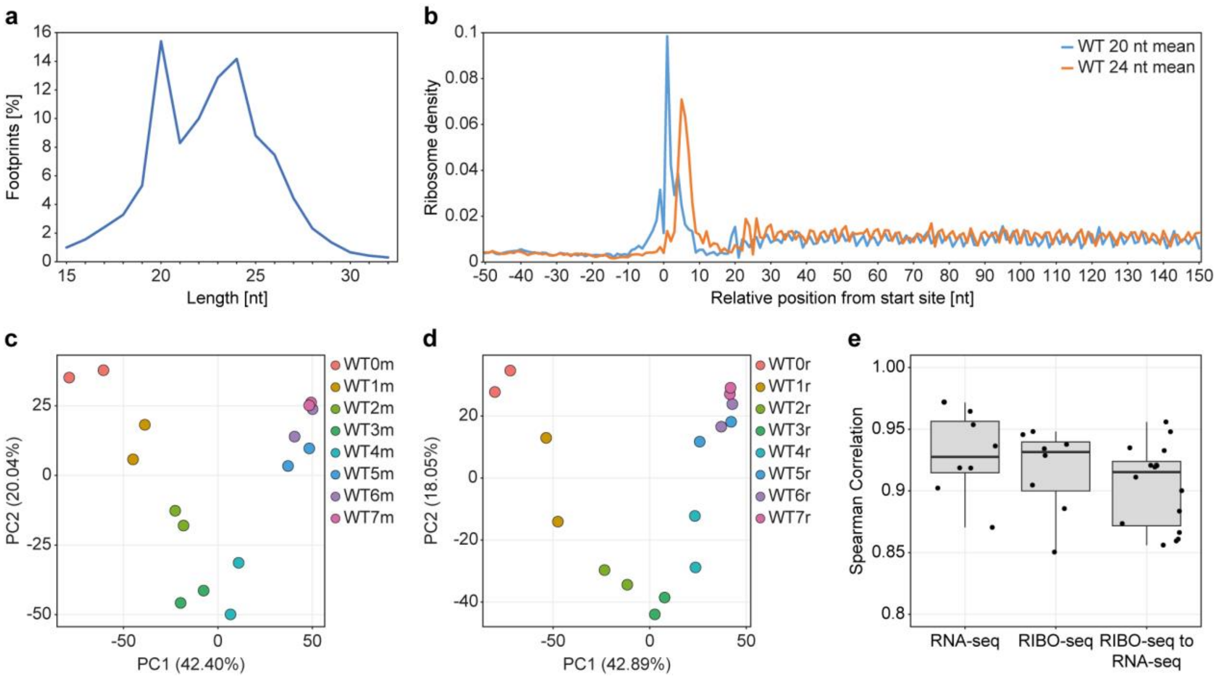


**Supplementary Fig. 1.** Quality Control of WT Data. (**a**) Length distribution of ribosomal footprints (RPFs) from all timepoints with duplicates. (**b**) Metagene plots of 3' assigned RPFs on ORF for 20 (blue) and 24 nt long (orange) footprints. (**c**) and (**d**) Principal component analysis plots of duplicate samples of the transcriptome (RNA-seq) and translatome (RIBO-seq) data for sporulating *Bacillus subtilis* at different timepoints: 0 to 7 hours post sporulation induction. (**e**) Distribution of Spearman's rho correlation values between duplicates for RNA-seq and RIBO-seq and between transcriptome (RNA-seq) and translatome (RIBO-seq) dataset pairs. Center line: median. Box: 25-75th percentiles (IQR). Whiskers: min/max values. Source data are provided as a Source Data file.

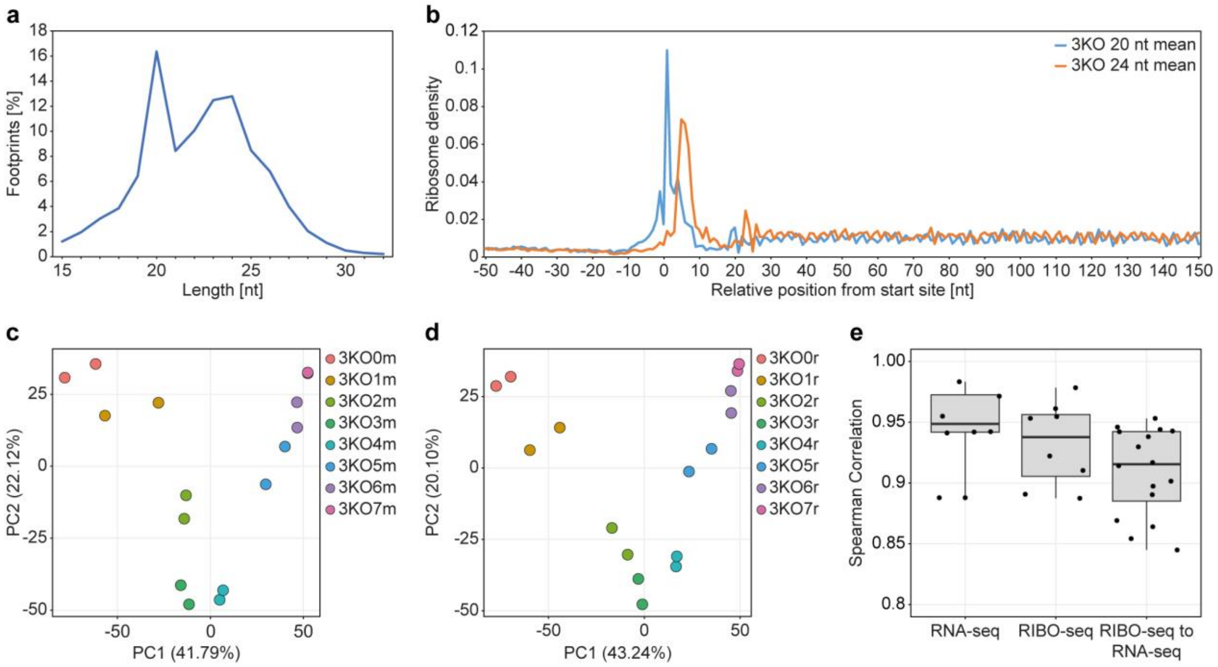


**Supplementary Fig. 2.** Quality Control of 3KO Data. (**a**) Length distribution of ribosomal footprints (RPFs) from all timepoints with duplicates. (**b**) Metagene plots of 3' assigned RPFs on ORF for 20 (blue) and 24 nt long (orange) footprints. (**c**) and (**d**) Principal component analysis plots of duplicate samples of the transcriptome (RNA-seq) and translatome (RIBO-seq) data for sporulating *Bacillus subtilis* at different timepoints: 0 to 7 hours post sporulation induction. (**e**) Distribution of Spearman's rho correlation values between duplicates for RNA-seq and RIBO-seq and between transcriptome (RNA-seq) and translatome (RIBO-seq) dataset pairs. Center line: median. Box: 25-75th percentiles (IQR). Whiskers: min/max values. Source data are provided as a Source Data file.

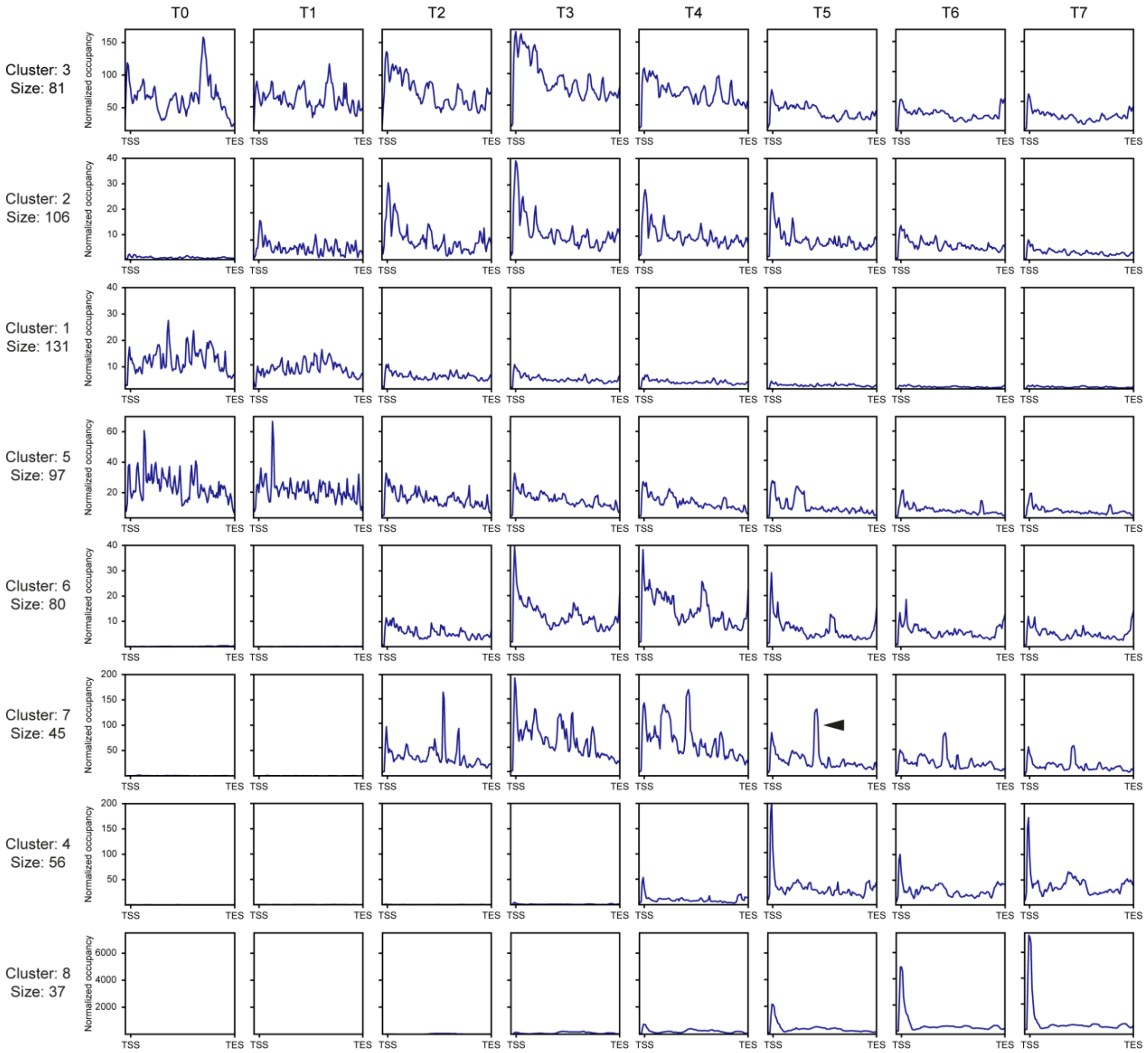


**Supplementary Fig. 3.** Mean ribosome density on CDSs with 5' UTR (-50 nt) during sporulation in *B. subtilis* from clusters presented in Figure 1. The X-axis shows the position in transcripts (TSS – transcript start site, TES – transcript end site). The Y-axis shows BPM (bins per million). Please note that there are different scales between clusters.

Arrowhead points to a peak resulting from ribosomes pausing on a single gene in cluster 7 – *cotE*. The ribosomes pause at rare codons, at the region involved in CotE localisation and oligomerisation [1]. Source data are provided as a Source Data file.

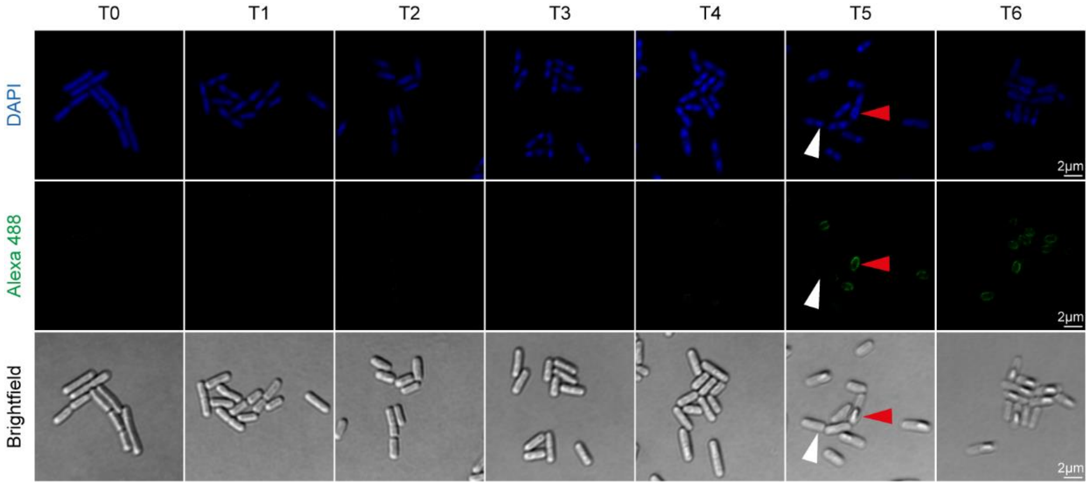


**Supplementary Fig. 4.** Microscopic images of *B. subtilis* WT cells treated with Alexa 488 (but not OPP) and DAPI during six hours of sporulation (T0 – T6). Phase bright spores are impermeable, and DAPI and Alexa staining is non-specific. Spores that completed engulfment but are not phase bright are permeable to the fluorescent dyes.

White arrowheads point to engulfed but not mature spore and show a lack of non-specific fluorescent labelling. The spore is permeable to fluorescent dye (DAPI inside the spore). Red arrowheads point to mature spore (PHASE BRIGHT) which is impermeable to fluorescent dyes and non-specifically labelled (spore coat labelled with DAPI and Alexa 488). Images of representative cells obtained from two biological replicates. The scale bar is 2 µm.

```
a
RpsN      MAKKSMIAKQQ--------------------------RTP--KFKVQEYTRCERCGRPHSVIRKFKLCRICFRELAYKGQIPGVKKASW
RpsNB     MAKKSKVAKELKRQQLVEQYAGIRRELKEKGDYEALSKLPRDSAPGRLHNRCMVTGRPRAYMRKFKMSRIAFRELAHKGQIPGVKKASW
          ***** :**:                           : *  .   : :.**   ***:: :****:.**.*****:************
b
RpmE      MKAGIHPNFKKATV---------------KCACGNEFETGSVKEEVRVEICSECHPFYTGRQKFASADGRVDRFNKKYGLK-
RpmEB     MKEGIHPKNHKVIFQDVNSGYRFLSTSTKTSNETAEWEDGNTYPVIKVEVSSDTHPFYTGRQKFNEKGGRVEQFKKRYNMGK
          ** ****: :*. .               ..    *:* *..   ::**:.*: ********** . .***::*:*:*.:
c
RpmGA     MRVNITLACTECGERNYISKKNKRNNPDRVEFKKYCPRDKKSTLHRETK
RpmGC     MRVNVTLACTETGDRNYITTKNKRTNPDRLELKKYSPRLKKYTLHRETK
          ****:****** *:****:.****.****:*:***.** ** *******
```

**Supplementary Fig. 5.** Alignment of paralogs of RpsN (S14), RpmE (L31), and RpmGA (L33) ribosomal proteins, respectively (**a**) RpsNB, (**b**) RpmEB, (**c**) RpmGC. The CXXC zinc-binding motif is marked in bold.

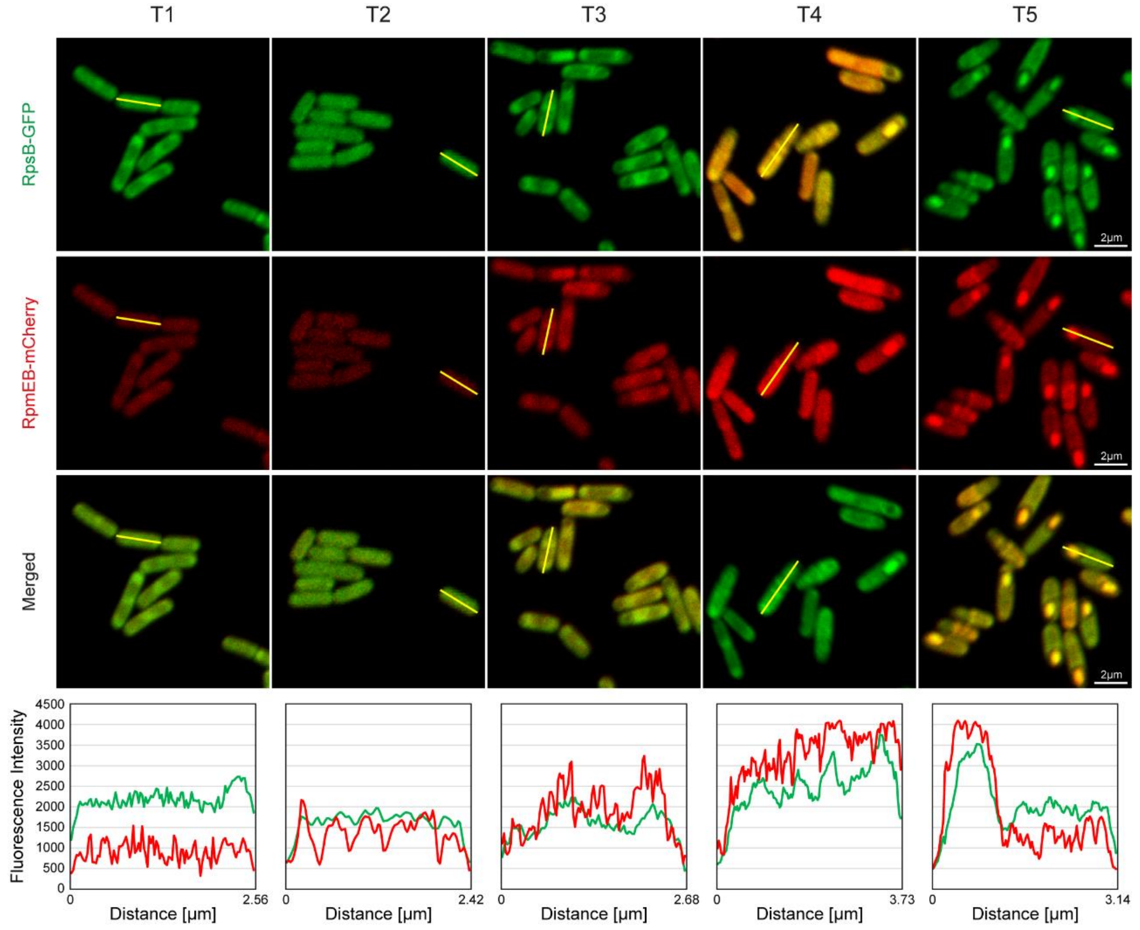


**Supplementary Fig. 6.** The ribosomal localisation of RpmEB at 1, 2, 3, 4, and 5 hours post-sporulation induction. The ribosomal proteins were tagged with fluorescent protein tags – RpsB-GFP (green) and RpmEB-mCherry (red) in the WT background.

The plots show the fluorescence intensity profiles measured along the yellow bars from the RpsB-GFP (green) and RpmEB-mCherry (red) images starting from a higher to a lower point. Peaks of mCherry fluorescence maxima correlate with the GFP fluorescence maxima at T3-T5 and correspond to the ribosomal localisation in the cell during sporulation. At T1 levels of mCherry fluorescence are lower than GFP, corresponding to lower expression of RpmEB during sporulation initiation, which then increases as sporulation progresses. Images of representative cells obtained from two biological replicates. The scale bar is 2 µm. Source data are provided as a Source Data file.

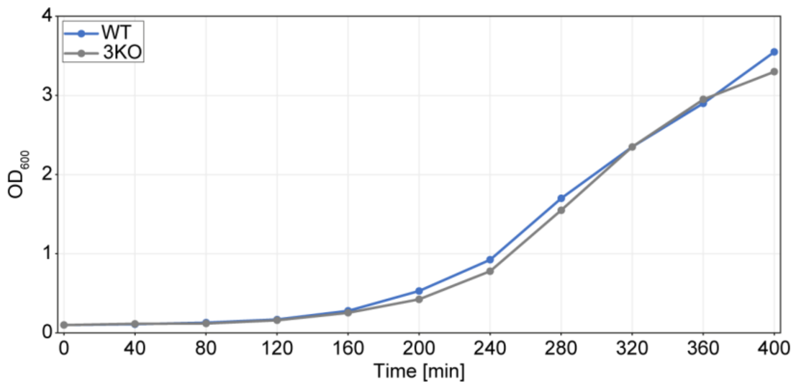


**Supplementary Fig. 7.** Growth Curves in CH Medium at 37°C for WT (blue) and 3KO (grey) Strains. Source data are provided as a Source Data file.

**Supplementary Table 1.** (**a**) Sporulation efficiency was calculated as the ratio between cells with asymmetric septum and cells without, for both WT and 3KO strains (n > 600), at 2, 3, and 4 hours post-sporulation induction; *indicates statistically significant differences (p-value<0.05) in sporulation efficiency between WT and 3KO, two-sided Fisher's test. **T2:** p-value = 0.04664, (95% confidence intervals: 0.9995484 1.6102781, odds ratio: 1.267723); **T3:** p-value = 1.727e-05, (95% confidence intervals: 1.379866 2.466235, odds ratio: 1.842484); **T4:** p-value = 0.3615, (95% confidence intervals: 0.6569939 1.1679672, odds ratio: 0.8772453). (**b**) The sporulation/germination efficiency was measured as the ratio of CFUs resulting from fully sporulated cultures before and after heat treatment (40 min at 90°C).

| **a. Sporulation efficiency at 2, 3, and 4 hours post-sporulation induction** | | | |
|---|---|---|---|
| **Strain** | **T2*** | **T3*** | **T4** |
| **WT** | 370/870 = 42% | 392/505 = 77% | 569/730 = 78% |
| **3KO** | 171/464 =37% | 318/487 = 65% | 415/518 = 80% |
| **b. Sporulation/germination efficiency before and after heat treatment** | | | |
| **Strain** | **$10^{-5}$** | **$10^{-5}$ heated** | **%** |
| **WT** | 160 | 101 | 63.1% |
| | 125 | 77.3 | 61.9% |
| | 142.3 | 83.3 | 58.5% |
| | 181.3 | 119 | 65.5% |
| | 160 | 94 | 58.7% |
| **3KO** | 212 | 37 | 17.4% |
| | 237 | 53.3 | 22.5% |
| | 161.7 | 71.7 | 44.3% |
| | 244 | 106.3 | 23% |
| | 267.6 | 61.6 | 43.6% |

**Supplementary Table 2.** Lists of (**a**) strains, (**b**) primers, (**c**) plasmids used in this study, and (**d**) adaptors and primers used to prepare cDNA libraries (from Multiplex Small RNA Library Prep Set for Illumina NEB #E7300 [2]).

**a. Strains**

| Strain | Genetic description | Reference |
|---|---|---|
| **WT** | *Bacillus subtilis* 168 | Burkholder and Giles, 1947 [3]; Spizizen, 1958 [4] |
| **Single KO** | *B. subtilis* 168 Δ*rpmEB* Kan$^R$ | This study |
| **2KO** | *B. subtilis* 168 Δ*rpmEB* Δ*rpsNB* Kan$^R$ Cm$^R$ | This study |
| **3KO** | *B. subtilis* 168 Δ*rpmEB* Δ*rpsNB* ΔrpmGC Kan$^R$ Cm$^R$ Ery$^R$ | This study |
| **WT-RpsB-GFP** | *Bacillus subtilis* 168 *rpsB*:*gfp* Sp$^R$ | This study |
| **3KO-RpsB-GFP** | *B. subtilis* 168 Δ*rpmEB* Δ*rpsNB* ΔrpmGC *rpsB*:*gfp* Kan$^R$ Cm$^R$ Ery$^R$ Sp$^R$ | This study |
| **WT-RpsB-GFP-RpmEB-mCherry** | *Bacillus subtilis* 168 *rpsB*:*gfp* Sp$^R$ *rpmEB:mCherry* Cm$^R$ | This study |

**b. Primers**

| Primer | Sequence |
|---|---|
| **UPFOR_rpmEB_1** | AGGAAGCACCAAAATTAAAATAGCAGG |
| **UPREV_rpmEB_1** | CACTGCCCGCTTTCCAGTCGGGGGGTATCTCCTTTCAATAAATCG |
| **MIDFOR_rpmEB_2** | CGATTTATTGAAAGGAGATACCCCCCGACTGGAAAGCGGGCAGTG |
| **MIDREV_rpmEB_2** | GACAAACCCTCAGGCCTGCCGTTATCGACAGCGGAATTGACTC |
| **DOWNFOR_rpmEB_3** | GAGTCAATTCCGCTGTCGATAACGGCAGGCCTGAGGGTTTGTC |
| **DOWNREV_rpmEB_3** | GCAGCATCGGCGTCTCCGACTTGGAC |
| **UPFOR_rpsNBcm_1** | ATTATCATTTTTGCAGTGGTTGGAAAGCTG |
| **UPREV_rpsNBcm_1** | CTTTATTATACAGATCTCCATGTCACATAGCCTCCCTTTAAATCG |
| **MIDFOR_rpsNBcm_2** | CGATTTAAAGGGAGGCTATGTGACATGGAGATCTGTATAATAAAG |
| **MIDREV_rpsNBcm_2** | CGGCGCGCTGTAATCGGCCGGAGTTTTTTCCACAAGAGGACGCTTTATTCTTCC |
| **DOWNFOR_rpsNBcm_3** | GGAAGAATAAAGCGTCCTCTTGTGGAAAAAACTCCGGCCGATTACAGCGCGCCG |
| **DOWNREV_rpsNBcm_3** | TCATTCTGGAAGGATATGCGAGAGCAATTG |
| **UPFOR_rpmGCe_1** | GGCGGCTCCATTGCAAGAAGGGCAACCATCTG |
| **UPREV_rpmGCe_1** | CCGTCTTATCTCCCATTATATCTTTTTTTTATTATACAACGTCATCACAAATTG |
| **MIDFOR_rpmGCe_2** | CAATTTGTGATGACGTTGTATAATAAAAAAAAGATATAATGGGAGATAAGACGG |
| **MIDREV_rpmGCe_2** | CGTAAAAAAAGACCGGGCCGTAAGGGAGTAGTATACCTAATAATTTATCTAC |
| **DOWNFOR_rpmGCe_3** | GTAGATAAATTATTAGGTATACTACTCCCTTACGGCCCGGTCTTTTTTTACG |
| **DOWNREV_rpmGCe_3** | GCGGTTCATTTGAGCAGTTTATATGACGGGAAAGAAATGACTTG |
| **UPFOR_rpsB_1** | GATACCTACGCCTCGTTTAGAATTCGCGGCGCAATC |
| **UPREV_rpsB_1** | CATTGATCCGCTGCCTGATCCGGACGCAGTTGTTGTTTCTGTTTC |
| **MIDFOR_rpsB-GFP_2** | GAAACAGAAACAACAACTGCGTCCGGATCAGGCAGCGGATCAATG |

| | |
|---|---|
| **MIDREV_rpsB-GFP_2** | GTATTTTTCCGTTAATCAAATTGCTCATTCACTTATAGAGTTCATCCATACC |
| **MIDFOR_rpsBsp_3** | GGTATGGATGAACTCTATAAGTGAATGAGCAATTTGATTAACGGAAAAATAC |
| **MIDREV_rpsBsp_3** | GTCCCTCTTATCACCTTTTGAATAGGTAATTGAGAGAAGTTTCTATAGAATTTTTC |
| **DOWNFOR_rpsB_4** | GAAAAATTCTATAGAAACTTCTCTCAATTACCTATTCAAAAGGTGATAAGAGGGAC |
| **DOWNREV_rpsB_4** | GTGTCTGCGCTCCGTAATATTCAACCGTTACTTTATCTAATAATG |
| **FOR rpmEB UP** | GCAGGAATGATAAAGCCGGAGATGCCGG |
| **REV rpmEB UP** | GTTATCCTCCTCGCCCTTGCTCACCATTTTCCCCATGTTATAGCGTTTTTTGAACTGC |
| **FOR rpmEB mCherry MID1** | GCAGTTCAAAAAACGCTATAACATGGGGAAAATGGTGAGCAAGGGCGAGGAGGATAAC |
| **REV mCherry cat MID1** | GTACATCCTTTCACAATTTGTCTACAGATTTTACTTGTACAGCTCGTCCATGCCG |
| **FOR mCherry cat MID2** | CGGCATGGACGAGCTGTACAAGTAAAATCTGTAGACAAATTGTGAAAGGATGTAC |
| **REV rpmEB cat MID2** | GACAAACCCTCAGGCCTGCCTTAGCACCTGTTATTGCAATAAAATTAGC |
| **FOR rpmEB DOWN** | GCTAATTTTATTGCAATAACAGGTGCTAAGGCAGGCCTGAGGGTTTGTC |
| **REV rpmEB DOWN** | GCAGCATCGGCGTCTCCGACTTGGAC |
| **c. Plasmids** | |
| **Name** | **Description** |
| **pAPNC-kan** | Used as a template for kanamycin resistance cassette |
| **pAPNC-erm** | Used as a template for erythromycin resistance cassette |
| **pAPNC-cm** | Used as a template for chloramphenicol resistance cassette |
| **pSHP2 [5]** | Used as a template for GFP and spectinomycin resistance cassette |
| **pMCL200** | Used as a template for mCherry and chloramphenicol resistance cassette |
| **d. Adaptors and primers used to prepare cDNA libraries** | |
| **Primer** | **Sequence** |
| **NEBNext SR Primer for Illumina** | AATGATACGGCGACCACCGAGATCTACACGTTCAGAGTTCTACAGTCCG*A |
| **NEBNext SR RT Primer for Illumina** | AGACGTGTGCTCTTCCGATCT |
| **NEBNext Index 1 Primer for Illumina** | CAAGCAGAAGACGGCATACGAGATCGTGATGTGACTGGAGTTCAGACGTGTGCTCTTCCGATC*T |
| **NEBNext Index 2 Primer for Illumina** | CAAGCAGAAGACGGCATACGAGATACATCGGTGACTGGAGTTCAGACGTGTGCTCTTCCGATC*T |
| **NEBNext Index 3 Primer for Illumina** | CAAGCAGAAGACGGCATACGAGATGCCTAAGTGACTGGAGTTCAGACGTGTGCTCTTCCGATC*T |
| **NEBNext Index 4 Primer for Illumina** | CAAGCAGAAGACGGCATACGAGATTGGTCAGTGACTGGAGTTCAGACGTGTGCTCTTCCGATC*T |
| **NEBNext Index 5 Primer for Illumina** | CAAGCAGAAGACGGCATACGAGATCACTGTGTGACTGGAGTTCAGACGTGTGCTCTTCCGATC*T |
| **NEBNext Index 6 Primer for Illumina** | CAAGCAGAAGACGGCATACGAGATATTGGCGTGACTGGAGTTCAGACGTGTGCTCTTCCGATC*T |
| **NEBNext Index 7 Primer for Illumina** | CAAGCAGAAGACGGCATACGAGATGATCTGGTGACTGGAGTTCAGACGTGTGCTCTTCCGATC*T |
| **NEBNext Index 8 Primer for Illumina** | CAAGCAGAAGACGGCATACGAGATTCAAGTGTGACTGGAGTTCAGACGTGTGCTCTTCCGATC*T |
| **NEBNext Index 9 Primer for Illumina** | CAAGCAGAAGACGGCATACGAGATCTGATCGTGACTGGAGTTCAGACGTGTGCTCTTCCGATC*T |
| **NEBNext Index 10 Primer for Illumina** | CAAGCAGAAGACGGCATACGAGATAAGCTAGTGACTGGAGTTCAGACGTGTGCTCTTCCGATC*T |

| | |
|---|---|
| **NEBNext Index 11 Primer for Illumina** | CAAGCAGAAGACGGCATACGAGATGTAGCCGTGACTGGAGTTCAGACGTGTGCTCT TCCGATC*T |
| **NEBNext Index 12 Primer for Illumina** | CAAGCAGAAGACGGCATACGAGATTACAAGGTGACTGGAGTTCAGACGTGTGCTCT TCCGATC*T |
| **NEBNext 3´ SR Adaptor for Illumina** | rAppAGATCGGAAGAGCACACGTCT-$NH_2$ |
| **NEBNext 5´ SR Adaptor for Illumina** | rGrUrUrCrArGrArGrUrUrCrUrArCrArGrUrCrCrGrArCrGrArUrC |

Where * indicates phosphorothioate bond

**Supplementary References:**


1. Little, Shawn, and Adam Driks. "Functional analysis of the Bacillus subtilis morphogenetic spore coat protein CotE." *Molecular microbiology* 42.4 (2001): 1107-1120.
2. NEBNext® Multiplex Small RNA Library Prep Set for Illumina® Set 1, Set 2, Index Primers 1–48 and Multiplex Compatible. Version 8.0_10/20. NEB. Accessed on: 23 March 2022. [Online]. Available: https://www.neb.com/media/nebus/files/manuals/manuale7300_e7330_e7560_e7580.pdf
3. Burkholder, P. R., and N. H. Giles. 1947. Induced biochemical mutations in *Bacillus subtilis*. Am. J. Bot. 34, 345-348.
4. Spizizen, J. 1958. Transformation of biochemically deficient strains of *Bacillus subtilis* by deoxyribonucleate. Proc. Natl. Acad. Sci. USA 44, 1072-1078.
5. Murina, V., Kasari, M., Takada, H., Hinnu, M., Saha, C. K., Grimshaw, J. W., ... & Atkinson, G. C. (2019). ABCF ATPases involved in protein synthesis, ribosome assembly and antibiotic resistance: structural and functional diversification across the tree of life. Journal of molecular biology, 431(18), 3568-3590.
