# Supplementary material for: Translation in Bacillus subtilis is spatially and temporally coordinated during sporulation
Source: Nat Commun. 2024 Aug 21;15:7188. doi: 10.1038/s41467-024-51654-6 (PMC11339384; doi:10.1038/s41467-024-51654-6)
Supplement: Supplementary file 3 — Description of Additional Supplementary Files [file 41467_2024_51654_MOESM3_ESM.pdf]

Description of Additional Supplementary Files:

Supplementary Data 1: List of genes from sigma factor regulons and sporulation genes from SubtiWiki. List of genes classified into clusters (Fig. 1a and b).

Supplementary Data 2: Statistics for mean AHA-Alexa 488 fluorescence intensities in WT and 3KO between timepoints with p-values (Fig. 3f and 5e).

Supplementary Data 3: Mass spectrometry results for the sucrose density gradientpurified monosome fractions of 3KO ribosomes compared to WT.

Supplementary Data 4: Differential Expression results from RIBO-seq data between 3KO and WT for all time points.

Supplementary Data 5: Summary of quality, trimming, mapping, and counting data from RNA-seq and RIBO-seq. Length distribution of ribosomal footprints from RIBO-seq.
