# Supplementary material for: Translation in Bacillus subtilis is spatially and temporally coordinated during sporulation
Source: Nat Commun. 2024 Aug 21;15:7188. doi: 10.1038/s41467-024-51654-6 (PMC11339384; doi:10.1038/s41467-024-51654-6)
Supplement: Supplementary file 9 — Reporting Summary [file 41467_2024_51654_MOESM9_ESM.pdf]

nature portfolio

Corresponding author(s): Agata L Starosta
Last updated by author(s): Jul 22, 2024

Reporting Summary

Nature Portfolio wishes to improve the reproducibility of the work that we publish. This form provides structure for consistency and transparency in reporting. For further information on Nature Portfolio policies, see our Editorial Policies and the Editorial Policy Checklist.

Statistics

For all statistical analyses, confirm that the following items are present in the figure legend, table legend, main text, or Methods section.

n/a	Confirmed	
☐	☒	The exact sample size (*n*) for each experimental group/condition, given as a discrete number and unit of measurement
☐	☒	A statement on whether measurements were taken from distinct samples or whether the same sample was measured repeatedly
☐	☒	The statistical test(s) used AND whether they are one- or two-sided<br>*Only common tests should be described solely by name; describe more complex techniques in the Methods section.*
☒	☐	A description of all covariates tested
☐	☒	A description of any assumptions or corrections, such as tests of normality and adjustment for multiple comparisons
☐	☒	A full description of the statistical parameters including central tendency (e.g. means) or other basic estimates (e.g. regression coefficient) AND variation (e.g. standard deviation) or associated estimates of uncertainty (e.g. confidence intervals)
☐	☒	For null hypothesis testing, the test statistic (e.g. *F*, *t*, *r*) with confidence intervals, effect sizes, degrees of freedom and *P* value noted<br>*Give P values as exact values whenever suitable.*
☒	☐	For Bayesian analysis, information on the choice of priors and Markov chain Monte Carlo settings
☒	☐	For hierarchical and complex designs, identification of the appropriate level for tests and full reporting of outcomes
☐	☒	Estimates of effect sizes (e.g. Cohen's *d*, Pearson's *r*), indicating how they were calculated

Our web collection on statistics for biologists contains articles on many of the points above.

Software and code

Policy information about availability of computer code

Data collection	Sequencing was performed as a service on an Illumina NextSeq500 by Genomed S.A. (Warsaw, Poland) and Genome Facility at CeNT (Warsaw, Poland); single-end 50bp reads were sequenced.<br><br>Confocal microscopy was carried out with an inverted confocal system Nikon C1.<br><br>Mass spectrometry was performed using UltiMate 3000 nano-LC system coupled to a Q Exactive HF-X mass spectrometer via an easy-spray source (all Thermo Fisher Scientific) as service.
Data analysis	Analyses of sequencing data were performed using: FastQC v.0.12.1, TrimGalore v.0.6.10, Bowtie v.1.3.1, STAR v.2.7.11b, sambamba v.1.0.1, featureCounts v.2.0.6, STATR, DeepTools v.3.5.5, DEBrowser v.1.30.2, R v.4.3.1 and DESeq2 library v.1.42.1.<br><br>Analyses of microscopic data were performed using: Fiji: ImageJ v.1.54f and R v.4.3.1.<br><br>Analyses of mass spectrometry data were performed using: MaxQuant v.1.6.17.0 and Perseus v. 1.6.10.0.

For manuscripts utilizing custom algorithms or software that are central to the research but not yet described in published literature, software must be made available to editors and reviewers. We strongly encourage code deposition in a community repository (e.g. GitHub). See the Nature Portfolio guidelines for submitting code & software for further information.

Data

Policy information about availability of data

All manuscripts must include a data availability statement. This statement should provide the following information, where applicable:

- Accession codes, unique identifiers, or web links for publicly available datasets
- A description of any restrictions on data availability
- For clinical datasets or third party data, please ensure that the statement adheres to our policy

The mass spectrometry proteomics data generated in this study have been deposited in the ProteomeXchange Consortium via the PRIDE partner repository under accession code PXD047497 [http://proteomecentral.proteomexchange.org/cgi/GetDataset?ID=PXD047497].

The RNA-seq and RIBO-seq data generated in this study have been deposited in the NCBI's Gene Expression Omnibus database under accession code GSE249450 [https://www.ncbi.nlm.nih.gov/geo/query/acc.cgi?acc=GSE249450].

Source data are provided with this paper.

Research involving human participants, their data, or biological material

Policy information about studies with human participants or human data. See also policy information about sex, gender (identity/presentation), and sexual orientation and race, ethnicity and racism.

Reporting on sex and gender	Not involved.
Reporting on race, ethnicity, or other socially relevant groupings	Not involved.
Population characteristics	Not involved.
Recruitment	Not involved.
Ethics oversight	Not involved.

Note that full information on the approval of the study protocol must also be provided in the manuscript.

Field-specific reporting

Please select the one below that is the best fit for your research. If you are not sure, read the appropriate sections before making your selection.

☒ Life sciences ☐ Behavioural & social sciences ☐ Ecological, evolutionary & environmental sciences

For a reference copy of the document with all sections, see nature.com/documents/nr-reporting-summary-flat.pdf

Life sciences study design

All studies must disclose on these points even when the disclosure is negative.

Sample size	For sequencing experiments, the number of samples was determined based on the length of the sporulation process, which is 7h in 37 Celsius degrees. Samples were taken in one hour intervals from T0 (logarithmic growth, just before sporulation initiation) up to T7 (seven hours post sporulation induction, mother cell lysis and spore release).<br>For TE and gene expression calculations, genes in appropriate sigma regulons were taken into consideration, unless stated otherwise, in which case all genes were used for analysis.<br>For microscopic observations, the exact numbers of cells for each experiment are given in the Materials and methods section.
Data exclusions	No data were excluded from the analyses.
Replication	All experiments were performed in duplicates, including sequencing, mass spectrometry and microscopy.<br>For mass spectrometry and sequencing experiments, the duplicates are described in the manuscript and all calculations are based on the entire dataset (both replicates). For microscopic observations, the experiments were performed in biological duplicates, however, data from one replicate is shown in the manuscript, as the biological repeat was successful and confirmed the observations from the first experiment.
Randomization	Randomization is not applicable in this study because there is no statistic test that requires randomization of samples.
Blinding	Samples were processed and analysed in exact same manner, thus, no blinding was required.

Reporting for specific materials, systems and methods

We require information from authors about some types of materials, experimental systems and methods used in many studies. Here, indicate whether each material, system or method listed is relevant to your study. If you are not sure if a list item applies to your research, read the appropriate section before selecting a response.

Materials & experimental systems

n/a	Involved in the study
☒	☐ Antibodies
☒	☐ Eukaryotic cell lines
☒	☐ Palaeontology and archaeology
☒	☐ Animals and other organisms
☒	☐ Clinical data
☒	☐ Dual use research of concern
☒	☐ Plants

Methods

n/a	Involved in the study
☒	☐ ChIP-seq
☒	☐ Flow cytometry
☒	☐ MRI-based neuroimaging

Plants

Seed stocks	*Report on the source of all seed stocks or other plant material used. If applicable, state the seed stock centre and catalogue number. If plant specimens were collected from the field, describe the collection location, date and sampling procedures.*
Novel plant genotypes	*Describe the methods by which all novel plant genotypes were produced. This includes those generated by transgenic approaches, gene editing, chemical/radiation-based mutagenesis and hybridization. For transgenic lines, describe the transformation method, the number of independent lines analyzed and the generation upon which experiments were performed. For gene-edited lines, describe the editor used, the endogenous sequence targeted for editing, the targeting guide RNA sequence (if applicable) and how the editor was applied.*
Authentication	*Describe any authentication procedures for each seed stock used or novel genotype generated. Describe any experiments used to assess the effect of a mutation and, where applicable, how potential secondary effects (e.g. second site T-DNA insertions, mosiacism, off-target gene editing) were examined.*
